# Correlation of a guidewire maximum insertion length with tortuous radial artery and the success rate during transradial coronary angiography

**DOI:** 10.1186/s12872-022-02933-x

**Published:** 2022-11-10

**Authors:** Hongsong Li, Xia Chen, Shuang Sha, Xiangdong Xu, Yingmin Chen

**Affiliations:** 1 Department of Cardiovascular Medicine, Jiading District Central Hospital Affiliated Shanghai University of Medicine and Health Sciences, Shanghai, 201800 China; 2 Clinical Research Center, Jiading District Central Hospital Affiliated Shanghai University of Medicine and Health Sciences, Shanghai, 201800 China; 3 Shanghai Key Laboratory of Molecular Imaging, Shanghai University of Medicine and Health Sciences, Shanghai, 201318 China; 4 Renji Hospital Shanghai Jiaotong University School of Medicine, Shanghai, China

**Keywords:** Radial artery, Coronary angiography, Guidewire

## Abstract

**Background:**

Percutaneous coronary intervention (PCI) is a safe and effective therapy for patients with obstructive coronary artery disease (CAD). We aimed to assess the correlation between the success rate of angiography and the maximum insertion length and resistance of a soft-tipped guidewire.

**Methods:**

Five hundred twenty-one patients were treated by successful radial artery puncture. According to whether the guidewire resistance, the patients were divided to three groups. 17 patients were maximum insertion length of guidewire ≤ 30 cm when resistance was encountered (group 1). 17 patients were maximum insertion length of guidewire between 30 and 45 cm when resistance was encountered (group 2). 487 patients were no resistance encountered (group 3).

**Results:**

The coronary angiography success rates of group 1, 2, and 3 were 52.94%, 47.05%, 98.97%, respectively. Typically, angiography can be completed in patients with Ω-shaped, S-shape or Z-shaped tortuosity.

**Conclusions:**

The maximum insertion length of straight guidewire and resistance can be used to determine radial artery status. The radial artery tortuosity or spasm significantly affects the success rate of coronary angiography.

## Background

In the past decade, transradial percutaneous coronary intervention (PCI) has become more and more popular, because it has fewer vascular complications, earlier ambulation, and more comfort [1–3]. The patients could walk immediately after operation, and the length of hospital stay was significantly shortened [4, 5]. Luovard and Lefevre suggested that the technical failures were mainly due to anatomical variations, such as radial loops or spastic, ectopic radial arteries, rather than subclavian tortuosity [6]. Byung-Su demonstrated that 4.2% of patients had a tortuosity of upper limb artery [7]. Most common site of vessel tortuosity as well as branching anomaly was proximal third of antecubital fossa. Therefore, retrograde angiography via radial sheath might be helpful in defining such a tortuosity. In advancing a guidewire or arterial sheath through a tortuous radial artery, technical difficulty and prolonged procedure time might be anticipated. There was a significant difference in procedure time and cross-over to femoral artery in patients with radial artery tortuosity on radial artery angiogram. Severe tortuosity configuration with an acute angle, such as the Ω-shape, α-shape and Z-like shape, were more related with procedural failure than less severe configuration, S-shape. An accurate knowledge of the normal or variant anatomy and tortuosity of the radial artery might be prerequisite for successful transradial coronary procedure [8–10]. However, it is difficult to evaluate the variant anatomy and tortuosity of radial artery before the insertion of arterial sheath. After successful puncture, we determined the condition of the radial artery and observe the success rate of transradial coronary angiography by means of maximum insertion length and resistance of a straight, soft-tipped guidewire.

## Methods

### The characteristics of participants

Patients who first received elective right transradial coronary angiography from January to December 2020. A total of 521 patients were enrolled. A written informed consent was taken from all patients after the explanation of the study. This study was approved by the ethics committee of Jiading District Central Hospital Affiliated Shanghai University of Medicine & Health Sciences (Shanghai, China). Inclusion criteria: (1) age > 18 years (2) the patients who were diagnosed as coronary heart disease or suspected coronary heart disease. (3) the patients did first percutaneous coronary angiography without any previous angiographic history via radial artery. (4) all enrolled patients signed informed consent. Exclusion criteria: (1) the patients with puncture failure or when the straight guidewire could not enter the lumen of the radial artery through the external cannula. (2) the patients whose systolic blood pressure difference of both upper limbs was more than 10 mm Hg. (3) the patients with radial artery dysplasia. (4) the patients with hepatic and renal insufficiency. (5) the patients with a history of fracture or deformity of the right upper limb.

## Puncture and cannulation

The ipsilateral limb was disinfected and draped. Lidocaine (0.5–1 ml, 2%) was used as local anesthesia. During injection of local anesthesia, attention was paid to the course of blood vessels to sufficiently block local tissues and prevent vasospasms. During blood vessel puncture, the wrist was overextended to sufficiently expose arteries. Bandages were also used as a cushion for the wrist to maintain wrist overextension. The puncture site was typically 2–3 cm near the transverse crease of the wrist and the ideal puncture point was found to be at least 10 mm proximal to the R-U line (the line between the styloid process and the ulnar styloid process) [11]. Catheterization at this site can avoid reticular tissues and small superficial branches. A cannula needle (TERUMO, Japan) was used to puncture the blood vessel. Pulsating backflow at the end of the needle was used to determine the relationship between the needle tip and blood vessel. After successful puncture, a 0.064 cm diameter and 45 cm long, straight, soft-tipped guidewire (TERUMO, Japan) was inserted along the puncture needle into the artery. Then, the guidewire was inserted to the proximal blood vessel. When resistance was encountered, a puncture needle external cannula was inserted into the radial artery along the straight guidewire and the guidewire was withdrawn. After pulsating backflow was present, a 10 ml syringe was used to inject onefold diluted contrast agent through the external cannula for retrograde angiogram of the forearm, elbow, and upper arm blood vessels. Anteroposterior imaging was selected. When overlapping blood vessel images were present, left anterior oblique or right anterior oblique 30° positions were used to determine blood vessel course. The seldinger technique was used to insert a 6F arterial sheath (TERUMO, Japan). When the guidewire encountered resistance, the sheath insertion depth was 1–2 cm lower than the maximum insertion depth of the guidewire. The number of punctures was no more than three times.

## Coronary angiography

2500 u of heparin and 200 μg of nitroglycerin were administered through the arterial sheath. Following that, a 0.089 cm diameter and 150 cm loach guidewire (TERUMO, Japan) and imaging catheter (Tig catheter, Goodman, China) were inserted through the arterial sheath into the radial artery, brachial artery, subclavian artery, brachiocephalic trunk and the aortic sinus. When the Tig catheter could be delivered to the sinus of the aorta but angiography could not be performed, a Judkins (3.5L/4R; Mindray, China) catheter may be attempted for angiography. In patients with a tortuous radial artery, a loach guidewire was used to straighten the artery as much as possible before the catheter was inserted. When the loach guidewire did not pass through the tortuous artery with its curved front end, we tried to shape the front end into a loop in the arterial sheath to improve the ability of passing through the tortuous vessels. If the guidewire or catheter could not pass through after multiple attempts, the tortuous radial artery could not be straightened, or when a vascular injury occurred, the arterial route was abandoned and the left radial or femoral artery was used for angiography. In all patients, the anatomy and possible injury of radial artery and brachial artery were evaluated by angiography before removing the radial artery sheath.

## Statistical processing

Quantitative data is expressed as mean ± standard deviation. Analysis of variance was used to compare quantitative data and a Chi-square test was used to compare qualitative data. A difference of *p* < 0.05 was considered statistically significant. All statistical analyses were conducted using SPSS 17 statistical software.

## Results

### Patient grouping and general information

According to whether the 45 cm length straight, soft-tipped guidewire resistance and the maximum insertion length in patients, the patients were divided into three groups (Table 1). 17 patients were maximum insertion length of guidewire ≤ 30 cm when resistance was encountered (group 1). 17 patients were maximum insertion length of guidewire between 30 and 45 cm when resistance was encountered (group 2). 487 patients were maximum insertion length of guidewire between 30 and 45 cm with no resistance encountered (group 3). The insertion length of guidewire was the distance between the head end of the wire and the intersection of the wire and the distal wrist crease.

**Table 1:** Comparison of general information of patients

Characteristic	Guidewire resistance		**Guidewire non-resistance**
	**Group 1**	**Group 2**	**Group 3**
**Number** **Age (y)**	17 68.88 ± 12.34	17 76.00 ± 8.48	487
			66.16 ± 11.62
**Male**	10	6	325
**Female**	7	11	161
**Height**	161.06 ± 8.61	159.65 ± 6.17	164.84 ± 7.31
**Weight**	63.47 ± 11.94	60.53 ± 11.36	66.86 ± 11.20
**Body mass index**	24.34 ± 3.61	23.63 ± 3.52	24.54 ± 3.43
**Smoking**	5	6	201
**Hypertension**	10	12	304
**Diabetes**	6	6	159
**Hyperlipidemia**	6	5	142
**Success rate**	9/ 17	8/ 17	482/ 487
**Vascular injury**	3	4	0

**Fig. 1:**
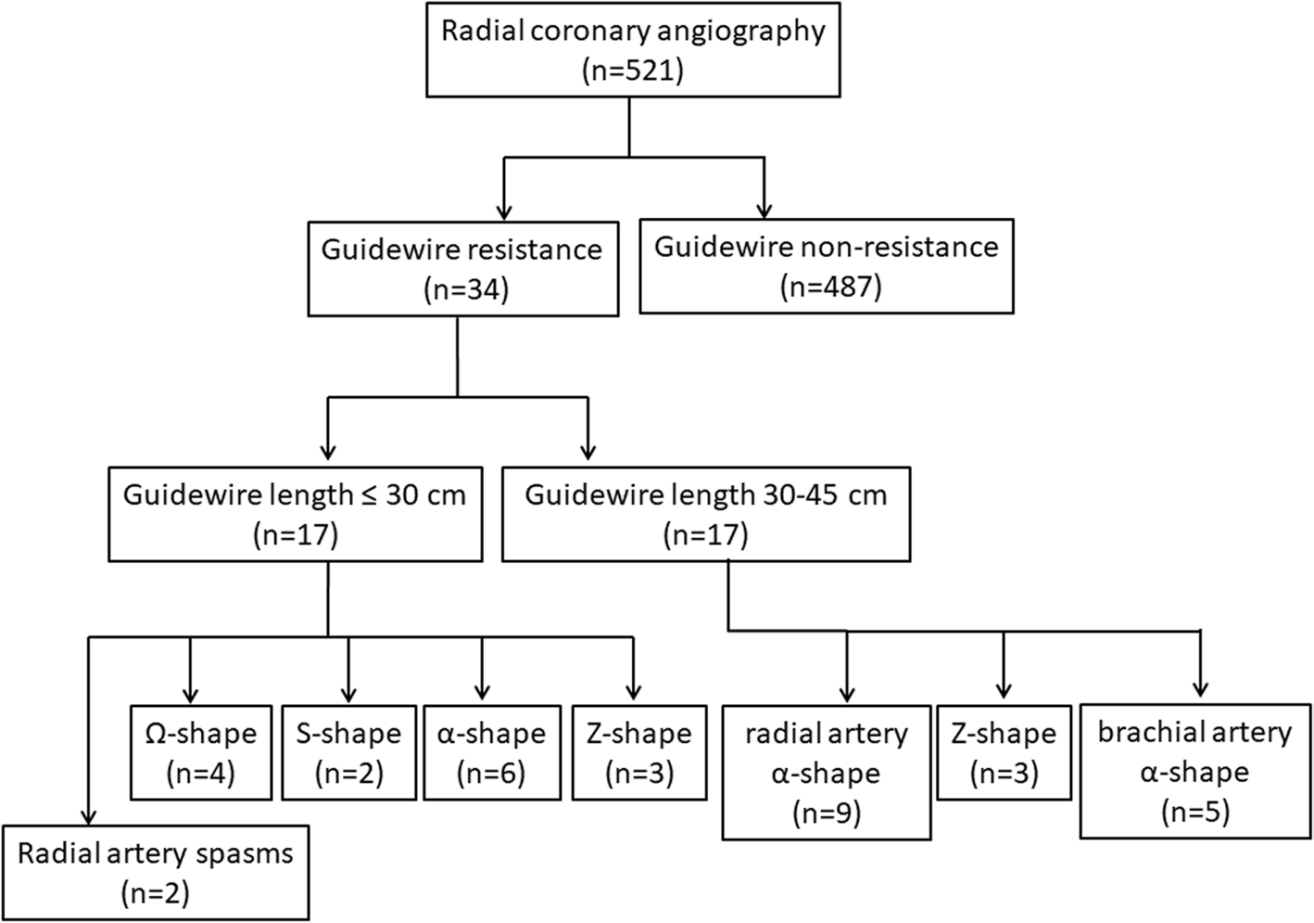
Flow diagram of radial coronary angiography of the patients

Of the 521 total patients, right transradial coronary angiography was successfully completed in 492 patients, resulting in an overall success rate of 94.43% (Fig. 1). Among the three groups, the ages of subjects in group 2 were significantly greater than in group 3 *(p* < 0.01 **), the heights of subjects in group 1 and group 2 were significantly lower than in group 3 *(p* < 0.05 *), the weight of subjects in group 2 were significantly lower than in groups 3 *(p* < 0.05 *), and the number of female patients was significantly greater in group 2 than in group 3 *(p* < 0.05 *). There were no differences in body mass index, smoking, hypertension, diabetes, or hyperlipidemia between the different groups. The right transradial coronary angiography success rates of groups 1, 2, and 3 were 52.94%, 47.05%, 98.97%, respectively. The success rate of group 3 was significantly higher than group 1 and group 2 (*p* < 0.01 **). During catheterization, the vascular injury incidence of group 3 was significantly lower than group 1 and group 2 (*p* < 0.01 **) (Table 2).

**Table 2:** Comparison of demographic profile in guidewire resistance and non-resistance groups

Characteristic	Guidewire resistance ≤ 30 cm	Guidewire non-resistance	*P*-value	Guidewire resistance 30–45 cm	Guidewire non-resistance	*P*-value
Ages	68.88 ± 12.34	66.16 ± 11.62		76.00 ± 8.48	66.16 ± 11.62	< 0.01**
Height	161.06 ± 8.61	164.84 ± 7.31	< 0.05*	159.65 ± 6.17	164.84 ± 7.31	< 0.05*
Weight	63.47 ± 11.94	66.86 ± 11.20		60.53 ± 11.36	66.86 ± 11.20	< 0.05*
Female	7	161		11	161	< 0.05*
Success rate	52.94%	98.97%	< 0.01**	47.05%	98.97%	< 0.01**
Vascular injury	3	0	< 0.01**	4	0	< 0.01**

## Patient radial arteriography

**Fig. 2:**
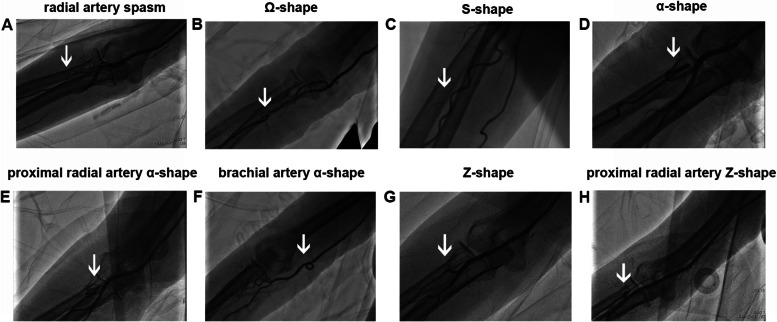
Retrograde angiogram showed the vessel tortuosity or spasms of upper limb artery (the radial artery or the brachial artery) when resistance of a straight, soft-tipped guidewire was encountered. **A** The radial artery spasms. **B** An acute angled (Ω-shape) of the radial artery. **C** A wavy tortuosity (S-shape) of the radial artery. **D** An annular tortuosity (α-shape) of the radial artery. **E** Annular tortuosity (α-shape) was present in the proximal segment of the radial artery and the small accessory brachial artery was present. **F** Annular tortuosity (α-shape) of the brachial artery. **G** Z-shaped tortuosity of the radial artery. **H** Z-shaped tortuosity was present in the proximal segment of the radial artery and the small accessory brachial artery was present

In the guidewire resistance length ≤ 30 cm group (group 1), the radial artery spasms occurred in 2 patients. An acute angled (Ω-shape) was present in the radial artery in 4 patients. A wavy tortuosity (S-shape) was present in the radial artery in 2 patients. An annular tortuosity (α-shape) was present in the proximal segment of the radial artery and the accessory brachial artery was absent in 6 patients, and Z-shaped tortuosity was present in the proximal segment of the radial artery in 3 patients. In patients with radial artery spasms, the catheter could not pass through the radial artery after cannulation and insertion of the loach guidewire. In patients with an acute angled (Ω-shape) or wavy tortuosity (S-shape) at the radial artery, the artery was straightened after cannulation and the imaging catheter passed through the radial artery for imaging. In patients with annular tortuosity (α-shape) present in the proximal segment of the radial artery, the loach guidewire could pass through the radial artery, but not the catheter. Imaging was successfully completed after cannulation in patients with Z-shaped tortuosity at the proximal segment of the radial artery (Fig. 2).

In the guidewire resistance length between 30 and 45 cm group (group 2), annular tortuosity (α-shape) was present in the proximal segment of the radial artery and the small accessory brachial artery was present in 9 patients, Z-shaped tortuosity was present in the proximal segment of the radial artery and the small accessory brachial artery was present in 3 patients, and annular tortuosity (α-shape) was present in the distal segment of the brachial artery in 5 patients. In the 5 patients with annular tortuosity (α-shape) in the distal segment of the brachial artery, the blood vessel was straightened after the loach guidewire was passed through the artery and imaging was successfully completed. In the 3 patients with Z-shaped tortuosity in the proximal segment of the radial artery, the guidewire successfully passed through the radial artery and imaging was completed. In patients with annular tortuosity (α-shape) in the proximal segment of the radial artery, the loach guidewire was unable to pass through the artery in 6 patients, while the loach guidewire could pass through the artery, but not the catheter in 3 patients.

In the guidewire non-resistance group (group 3), imaging was not completed in 5 patients because the right subclavian artery or the brachiocephalic trunk was extremely tortuous. Imaging was successfully completed in the remaining patients.

## Discussion

The transradial route is preferred for coronary angiography due to lower trauma, fewer bleeding complications, and no need to restrict patient movement [1–3]. However, the condition of upper limb blood vessels, particularly the radial artery, is an important factor limiting surgery success [6, 7, 12].

The common reasons for transradial coronary angiography failure include: 1) puncture failure; 2) the guidewire and catheter can’t pass through for the next step after vascular access is achieved; 3) the imaging device could enter the aortic root, but the catheter could not be inserted into position; and 4) forceful imaging resulting in unclear or incomplete blood vessels [13, 14]. In a study by He et al., the above determination criteria were used. Among subjects in the statistical analysis, imaging was successful in 938 patients and failure occurred in 109 patients, resulting in a success rate of 89.59%. The authors attributed the primary reason for failure to be anatomical abnormalities in the radial artery, including vascular stenosis, tortuosity, and dysplasia. This accounted for the failure observed in 56 patients, the percent rate of failure was 51% [1]. Of all 521 patients, right transradial coronary angiography was successful in 492 patients, resulting in an overall success rate of 94.43%. However, we did not include puncture failure patients. The failure reasons for all the 521 patients can be attributed to the guidewire and catheter could not pass through for the next step after vascular access is achieved. The results indicate that upper limb blood vessel condition, particularly radial artery condition, is an important factor for coronary procedures success for experienced surgeons after successful radial artery puncture. We divided the patients into three groups based on the maximum insertion length and resistance of a straight, soft-tipped guidewire. Results showed that the coronary angiography success rate in group 1 and group 2 were significantly lower than group 3. This shows that straight guidewire resistance can predict transradial coronary angiography success rate.

We performed angiography in conjunction with a measure of straight guidewire resistance to determine the arterial condition. The incidence of severe radial artery spasms was occurred, requiring a change in the approach. Vasospasms often worsen after cannulation and cause pain, which affects further operation. However, imaging was successfully completed after cannulation in patients with Ω-shaped, and S-shaped tortuosity in radial artery. Imaging could not be completed in patients with annular tortuosity (α-shape) in the proximal segment of the radial artery and variation (e.g., the small accessory brachial artery converged with the middle and upper segments of the brachial artery), but imaging could be completed in patients with Z-shaped tortuosity in the proximal segment of the radial artery and annular tortuosity (α-shape) in the distal segment of the brachial artery. In guidewire non-resistance group (group 3), the main causes of failure were tortuous right subclavian artery or brachiocephalic trunk, and the catheter could not enter coronary artery opening. Failure patients in group 3 could not be predicted using the straight guidewire. The incidence of annular tortuosity (α-shape) in the proximal segment of the radial artery was high in failure patients in group 1 and group 2. However, the difference was that a small accessory brachial artery was not present at the top of the annular tortuosity in group 1, while the accessory brachial artery that was parallel to the brachial artery was often present in group 2. This is the reason why the guidewire could not be inserted deeply in group 1, while the guidewire could be inserted deeply in group 2. This is because the guidewire could enter the accessory brachial artery along the tortuous top. As these blood vessels are fine, significant resistance is often present. If tortuosity is absent, the guidewire can usually reach the proximal segment of the brachial artery and resistance is absent. This was what was observed in group 3. Additionally, due to the tortuosity of blood vessels in group 1 and group 2, the operation of loach wire or catheter becomes complicated and time-consuming, and the risk of vascular injury is also increased.

Further analysis found that the ages of subjects in group 2 were significantly greater than in group 3 (*p* < 0.01**), the heights of subjects in group 1 and group 2 were significantly lower than in group 3 (*p* < 0.05*), the weight of subjects and the number of female patients in group 2 were significantly lower than in group 3 *(p* < 0.05*), Ostojić Z reported that radioulnar loops, being one of the potential contraindications for the procedure, were reported in 2% of cases [15]. Regression analysis revealed that age, female sex and high origin considerably contributed to the development of tortuosity. This suggests that attention should be paid to vascular status, particularly for thin, elderly, and female patients.

When selecting the transradial approach for coronary angiography, vascular access is a key to determining the success of the procedure [16, 17]. Puncture success rate is associated with the surgeon's experience and preoperative feeling of radial artery pulsation, which can be used to assess the possibility of puncture success. There is less experience on how to predict the condition of the radial artery after successful puncture. If the puncture cannula is blindly inserted, this may damage the radial artery. If severe tortuosity is present in the radial artery, the guidewire and imaging catheter cannot pass through the artery. If the guidewire and catheter are forcefully inserted, this tends to cause vasospasms, vascular injury, or even rupture, increasing patient suffering. In the current study, the maximum insertion length of the guidewire and resistance were used to determine the condition of the radial artery after successful puncture. Upper limb angiography was immediately conducted when resistance was encountered to understand the condition of the radial artery and prevent blind operation. This lessened the possibility of suffering and vascular injury in patients. The study also had limitations. Though patients with puncture failure have been excluded, we did not record the number of radial artery punctures. Multiple punctures may cause spasm at the distal end of the radial artery, which may be increase the resistance when the guidewire enters. Moreover, there is a large difference in the number of cases in different groups, which may lead to selective bias.

## Conclusions

In this study, we conclude that the maximum length and resistance of a straight guidewire can be used to determine radial artery status. The resistance of guidewire is considered that the presence of radial artery spasms, or tortuosity, all of which significantly affect the success rate of coronary angiography. When the guidewire resistance was occurred, puncture needle external cannula imaging can be used to determine the course of the radial artery. The course should be changed in patients with severe spasms, or annular tortuosity. Angiography can typically be completed in patients with Ω-shaped, S-shaped, or Z-shaped tortuosity. In this study, the balloon-assisted tracking (BAT) technique was not used to insert the imaging catheter. The BAT technique can further raise the catheter through the spasmodic radial artery and increase the success rate of coronary procedures [18].

## Data Availability

The datasets used and/or analyzed during the current study are available from the corresponding author on reasonable request.

## References

[1] Sachdeva S, Saha S (2014). Transradial approach to cardiovascular interventions: an update. Int J Angiol.

[2] Sandhu K, Butler R, Nolan J (2017). Expert Opinion: Transradial Coronary Artery Procedures: Tips for Success. Interv Cardiol.

[3] Jolly SS, Yusuf S, Cairns J, Niemela K, Xavier D, Widimsky P, Budaj A, Niemela M, Valentin V, Lewis BS (2011). Radial versus femoral access for coronary angiography and intervention in patients with acute coronary syndromes (RIVAL): a randomised, parallel group, multicentre trial. Lancet.

[4] Yoon JHLS, Lee HH, Kim JY, Hwang SO, Choe KH (1998). The experience of trans-radial coronary intervention in Wonju. Korean Circ J.

[5] Goldberg SL, Renslo R, Sinow R, French WJ. Learning curve in the use of the radial artery as vascular access in the performance of percutaneous transluminal coronary angioplasty. Cathet Cardiovasc Diagn. 1998;44(2):147-52. 10.1002/(sici)1097-0304(199806)44:2<147::aid-ccd5>3.0.co;2-6 9637436

[6] Louvard Y, Lefevre T (2000). Loops and transradial approach in coronary diagnosis and intervention. Catheter Cardiovasc Interv.

[7] Yoo BS, Yoon J, Ko JY, Kim JY, Lee SH, Hwang SO, Choe KH (2005). Anatomical consideration of the radial artery for transradial coronary procedures: arterial diameter, branching anomaly and vessel tortuosity. Int J Cardiol.

[8] Yokoyama N, Takeshita S, Ochiai M, Koyama Y, Hoshino S, Isshiki T, Sato T (2000). Anatomic variations of the radial artery in patients undergoing transradial coronary intervention. Catheter Cardiovasc Interv.

[9] Drizenko A, Maynou C, Mestdagh H, Mauroy B, Bailleul JP (2000). Variations of the radial artery in man. Surg Radiol Anat.

[10] Jurjus A, Sfeir R, Bezirdjian R (1986). Unusual variation of the arterial pattern of the human upper limb. Anat Rec.

[11] Fujii T, Masuda N, Toda E, Shima M, Tamiya S, Ito D, Matsukage T, Ogata N, Morino Y, Tanabe T (2010). Analysis of right radial artery for transradial catheterization by quantitative angiography–anatomical consideration of optimal radial puncture point. J Invasive Cardiol.

[12] Dahm JB, van Buuren F (2010). Transradial percutaneous coronary interventions: technique, materials & procedure in the light of anatomical and technical considerations. Indian Heart J.

[13] Norgaz T, Gorgulu S, Dagdelen S (2012). A randomized study comparing the effectiveness of right and left radial approach for coronary angiography. Catheter Cardiovasc Interv.

[14] Riangwiwat T, Mumtaz T, Blankenship JC (2020). Barriers to use of radial access for percutaneous coronary intervention. Catheter Cardio Inte.

[15] Ostojic Z, Bulum J, Ernst A, Strozzi M, Maric-Besic K (2015). Frequency of radial artery anatomic variations in patients undergoing transradial heart catheterization. Acta Clin Croat.

[16] Valsecchi O, Vassileva A, Musumeci G, Rossini R, Tespili M, Guagliumi G, Mihalcsik L, Gavazzi A, Ferrazzi P (2006). Failure of transradial approach during coronary interventions: anatomic considerations. Catheter Cardiovasc Interv.

[17] Scalise RFM, Salito AM, Polimeni A, Garcia-Ruiz V, Virga V, Frigione P, Ando G, Tumscitz C, Costa F. Radial Artery Access for Percutaneous Cardiovascular Interventions: Contemporary Insights and Novel Approaches. J Clin Med. 2019;8(10):1727-46. 10.3390/jcm8101727 PMC6833028 31635342

[18] Wojciuk J, Beijk MA, Goode G, Brack M, Galasko G, More R, Roberts D, Eichhofer J, Patel B, Chauhan A (2019). Balloon-assisted tracking technique as 'a way forward' for transradial intervention. Coron Artery Dis.

